# Optical fiber-based dispersion for spectral discrimination in fluorescence lifetime imaging systems

**DOI:** 10.1117/1.JBO.25.1.014506

**Published:** 2019-12-12

**Authors:** Md Abdul Kader Sagar, Bing Dai, Jenu V. Chacko, Joshua J. Weber, Andreas Velten, Scott T. Sanders, John G. White, Kevin W. Eliceiri

**Affiliations:** a University of Wisconsin–Madison, Laboratory for Optical and Computational Instrumentation, Madison, Wisconsin, United States; b University of Wisconsin–Madison, Biomedical Engineering Department, Madison, Wisconsin, United States; c University of Wisconsin–Madison, Mechanical Engineering Department, Madison, Wisconsin, United States; d University of Wisconsin–Madison, Medical Physics Department, Madison, Wisconsin, United States; e Morgridge Institute for Research, Madison, Wisconsin, United States

**Keywords:** hyperspectral imaging, multiphoton microscopy, fiber-based dispersion, time-correlated single-photon counting, fluorescence lifetime imaging, fluorescence lifetime

## Abstract

The excited state lifetime of a fluorophore together with its fluorescence emission spectrum provide information that can yield valuable insights into the nature of a fluorophore and its microenvironment. However, it is difficult to obtain both channels of information in a conventional scheme as detectors are typically configured either for spectral or lifetime detection. We present a fiber-based method to obtain spectral information from a multiphoton fluorescence lifetime imaging (FLIM) system. This is made possible using the time delay introduced in the fluorescence emission path by a dispersive optical fiber coupled to a detector operating in time-correlated single-photon counting mode. This add-on spectral implementation requires only a few simple modifications to any existing FLIM system and is considerably more cost-efficient compared to currently available spectral detectors.

## 1 Introduction

Fluorescence lifetime imaging (FLIM) microscopy is a microscopy technique that maps the fluorescence lifetime values at each voxel (the average time spent by the molecule in the excited state) into image contrast. FLIM can reveal spatial variations in the microenvironment of a sample by the virtue of the molecule’s available electronic states and the relaxation times from those levels to its ground state.^1^ The technique of molecular probing using fluorescence lifetime has enabled the development of optical methods that reveal a wide range of properties, including molecular binding activity, and autofluorescence-based diagnostics.^2^ Another fluorescence methodology that can provide information on the identity and microenvironment of a molecule is spectral or, when implemented across a broader sensing range, hyperspectral imaging (HSI).^3^

FLIM measures the fluorescence intensity as a function of time between excitation and fluorescence emission. Time-domain FLIM acquisition methods use high time-resolution electronics to measure the arrival time of the emission photon relative to the time of excitation photon pulse. Fluorescence is a stochastic process; many individual photon events must be measured to characterize the lifetime of a fluorophore. The minimum number of events needed to accurately determine the fluorescence lifetime limits the speed of FLIM. HSI is an imaging technique that maps the fluorescence emission spectrum as a false color image.^4^ These techniques are well-established and are currently used in a wide range of applications from food and dietary sciences^5^ to semiconductor nanocrystals studies and quantum dots.^6^ The simultaneous detection of spectral and lifetime information provides extra dimensions of data from fluorescence signals which can be used to facilitate the identification of a fluorophore. These environmentally sensitive fluorescence parameters can determine aspects of the molecule’s physical and chemical association with other molecules.

With a combined FLIM-HSI correlative microscopy scheme, each dimension (spectrum and lifetime) is simultaneously acquired. However, when multiple fluorophores are present in the sample, the resulting histogram of emission events is no longer a simple exponential decay or a single emission spectrum. The events from multiple fluorophores are combined (both spectrally and temporally). To separate different species of fluorescence emission, spectral methods, such as linear unmixing^7^ or deconvolution,^8^ can be used for HSI, and phasor analysis^9^ and multiexponential fitting can be used for FLIM. However, these deconvolution approaches grow more complicated with increasing numbers of fluorophores (i.e., with an increasing number of contributions from different exponentials), and thus the approach is limited to a small number of spectral components. However, these methods have been successful in computationally deconvolving the overlapping curves to separate up to seven independent species.^10^^–^^13^ Recently, correlative species identification schemes that use both spectral and lifetime information have been introduced.^14^^,^^15^

Multispectral FLIM is implemented currently using one of the following strategies. Samples can be imaged multiple times, each time using a different optical bandpass filter. However, acquiring multiple images is generally undesirable, as it is time-consuming, and multiple exposures increase the risk of photobleaching or otherwise damaging the sample. This approach can also be implemented as simultaneous imaging of two or more channels by splitting the emission spectra into multiple fixed spectral bandwidth channels by dichroic mirrors and filters, with each channel sharing the timing electronics by intelligent routing. Roberts et al.^16^ implemented a four-channel time-correlated single-photon counting (TCSPC) system, where each channel has an individual photomultiplier tube (PMT). Another option is to simultaneously measure all of the spectrum using a spatially dispersive optical element, such as a prism^17^ or a grating,^18^^,^^19^ and directing the emission on to multiple timing detectors. The dispersive element and multiple detectors, however, make this a technically complicated and expensive solution. Following the line of reasoning to use dispersive elements in the optical path for spectral separation, instead of using an optical prism or optical grating to introduce dispersion, one could use an optical fiber to achieve chromatic dispersion. Effective utilization of dispersive properties of optical fibers has been demonstrated in various optical communication applications, such as wavelength-division optical multiplexing^20^ and optical time-domain reflectance characterization.^21^ By specifying the length and choosing the right material, the optical fiber can introduce appropriate dispersion to allow the separation of the fluorescence emission spectrum to the desired resolution. This approach forms the basis of this study.

Prior studies demonstrating spectral imaging using optical fibers as the dispersive element have been reported in the literature. Sun et al.^22^ demonstrated a novel fiber optics-based method for simultaneous time- and wavelength-resolved fluorescence spectroscopy by combining three sets of bandpass filters and dichroic mirrors in a single acquisition. Three channels were coupled by optical fibers to introduce temporal delays. This approach was extended by Shrestha et al.^23^ to integrate the scheme in a scanning multispectral FLIM system. The dichroic mirror and filters were chosen such that it can separate emission spectra of three fluorophores to three channels. Optical fibers of different lengths were used to introduce distinct temporal delays for each channel, thus allowing for multichannel spectral FLIM (sFLIM) with a single detector, a significant step up from the multidetector approach.^17^ Nevertheless, this approach replies on the combination of filters, dichroic mirrors, and optical fibers to achieve sFLIM, thus requiring considerable modifications to a conventional multiphoton microscope. Also, in these systems, the optical fibers were used to route the signal rather than for their dispersive properties. However, a fiber-based multichannel that used this setup was used to detect glycosaminoglycan loss in articular cartilage.^24^ The utilization of dispersive property of optical fiber has been used to generate a rapid excitation scan. Rapid wavelength scan of laser-induced fluorescence was demonstrated by transmitting broadband excitation light through a mile-long optical fiber which introduced group-velocity (i.e., spectral) dispersion.^25^ Goda et al.^26^ used a serial time-encoded amplified microscopy camera along with an optical fiber to effectively map a two-dimensional (2-D) spatial image into a serial time-domain data stream for ultrafast real-time optical imaging. However, this does not work with low-intensity signals, such as fluorescence emission, and gives no spectral information on the specimen. Redding et al.^27^^,^^28^ demonstrated a high-resolution spectrometer by reconstructing an arbitrary spectrum from the output intensity profile recorded by a 2-D camera, based on precalibrated wavelength-dependent speckle patterns produced by interference between the guided modes of a multimode optical fiber. The idea was extended by integrating a wavelength-division multiplexer with seven multimode fibers to increase the spectral bandwidth.^20^ While the subnanometer resolution was achieved along with 100-nm bandwidth, this technique lacks lifetime imaging capability. One study^29^ has demonstrated utilizing dispersive property of optical fiber to perform Raman spectroscopy without spectrometer. This fiber-based approach was extended^30^ using superconducing nanowire single-photon detector for higher temporal accuracy.

In this paper, we propose a simple, cost-efficient method that, by the addition of an optical fiber to a conventional single detector multiphoton FLIM microscope, to obtain spectral information of emission signals which is otherwise unavailable without the fiber in addition to the lifetime information. This method will work with any multiphoton or confocal microscope with a pulsed excitation scheme and TCSPC electronics. While it is demonstrated using time-domain single-photon counting, this method is extendable to other systems that are capable of measuring lifetime. The method proposed here produces additional spectral information together with the lifetime distribution within the field of view (FOV). We first present the theory and rationale, then describe the experimental setup, and report the optimizations obtained with an appropriate choice of commercially available optical fibers. The measurements are validated, and data analysis techniques to map spectral separation are provided to enable the extension of the imaging capabilities of an FLIM system to combined HSI-FLIM. Proof of principle experiments are demonstrated where spectral and lifetime information are extracted from signals from fluorescent beads and fluorescently labeled cells. The impact of the technique and its potential are finally discussed.

## 2 Theory

**Fig. 1:**
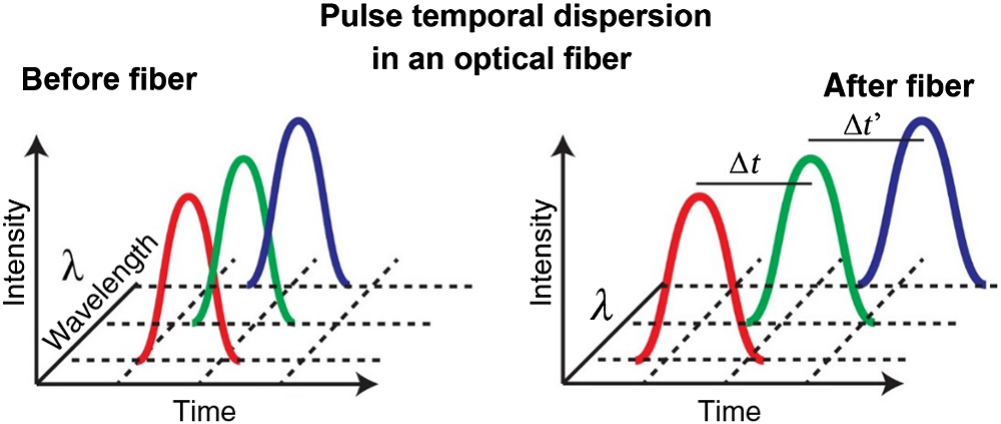
Spectral separation achieved by virtue of the chromatic dispersion of an optical fiber.

Generally, optical chromatic dispersion is used to separate different wavelengths in spectrally resolved lifetime imaging systems. However, instead of relying on spatial dispersion to send different wavelengths of light to different detectors (such as with a prism or grating), temporal chromatic dispersion introduced by an optical fiber is used to separate all the wavelengths and send them to a single detector in sequence (refer Fig. 1).

Spectral dispersion is achieved by guiding the emission fluorescence signal through a multimode optical fiber, which introduces different travel times for different wavelengths of light that travel through the fiber, i.e., chromatic dispersion. The red (longer wavelength) components of the fluorescence, which see a lower index of refraction, travel faster through the fiber than the blue (shorter wavelength). Thus, the spectral information of the fluorescence is encoded into the timing of the photon arrival at the detector. Once the decay curves of fluorophores at each pixel are recorded by TCSPC, computational deconvolution of the decay curves allows one to separate individual decay curves from different colored fluorophores. This deconvolution is practically limited by the finite instrument response function (IRF) of the photon detector (Commercial TCSPC systems vary from 100 to 350 ps IRF with a time resolution of ∼40  ps). The spectral information content per pixel is convolved with the lifetime histogram, which in turn is convolved with the shape of the IRF. Hence, the average spectral information can be deduced as the average relative time delay (or shift) of the leading edge of each fluorescence decay curve. In this manner, with the knowledge of the dispersion characteristics of the fiber such as the Sellmeier coefficients of the material of the fiber, attenuation, and bandwidth, it is possible to determine the mean wavelength of a photon distribution encoded in the transit delay through the fiber without deconvoluting the decay curves.

If the spectral dispersion in the fiber is sufficient, there is enough time difference between the arrival times of the different colors of light at the detector that the fluorescence decay curves for the individual fluorophores can be separated. The temporal dispersion experienced by the emission light in the optical fiber is a consequence of the wavelength-dependent index of refraction. The time delay for a light pulse (assuming plane wave) propagating through an optical fiber of length L is L(n−λdn/dλ)/c, where c is the speed of light in vacuum. The speed of light in a medium, v, is then approximated as $v=c/n_{\lambda }$, where $n_{\lambda }$ is the wavelength-dependent group refractive index. Therefore, the transit time difference through an optical fiber for light of two different wavelengths can be written as

$$\Delta t=L/v_{\lambda 1}-L/v_{\lambda 2}\;\Delta t=L(n_{\lambda 1}-n_{\lambda 2})/c\;\Delta t=L\Delta n_{\lambda }/c.$$ (1)

For common optical glasses, the index of refraction variation over the visible region of the spectrum is small, generally <1%.^31^ For example, for fused silica (a commonly used material for glass optical fiber fabrication), group refractive indices of n=1.4623 at 500 nm and 1.4618 at 510 nm are reported. With fast detectors and timing electronics with a resolution of 50 ps, in order to achieve 10-nm spectral resolution, we will need a fiber length of 30 m. In our proposed experiment, we, therefore, adopt optical fibers of at least 10-m long for the sake of sufficient spectral separation. More detailed analysis is presented in Sec. 4.1.

## 3 Methods

### 3.1 Experimental Setup

**Fig. 2:**
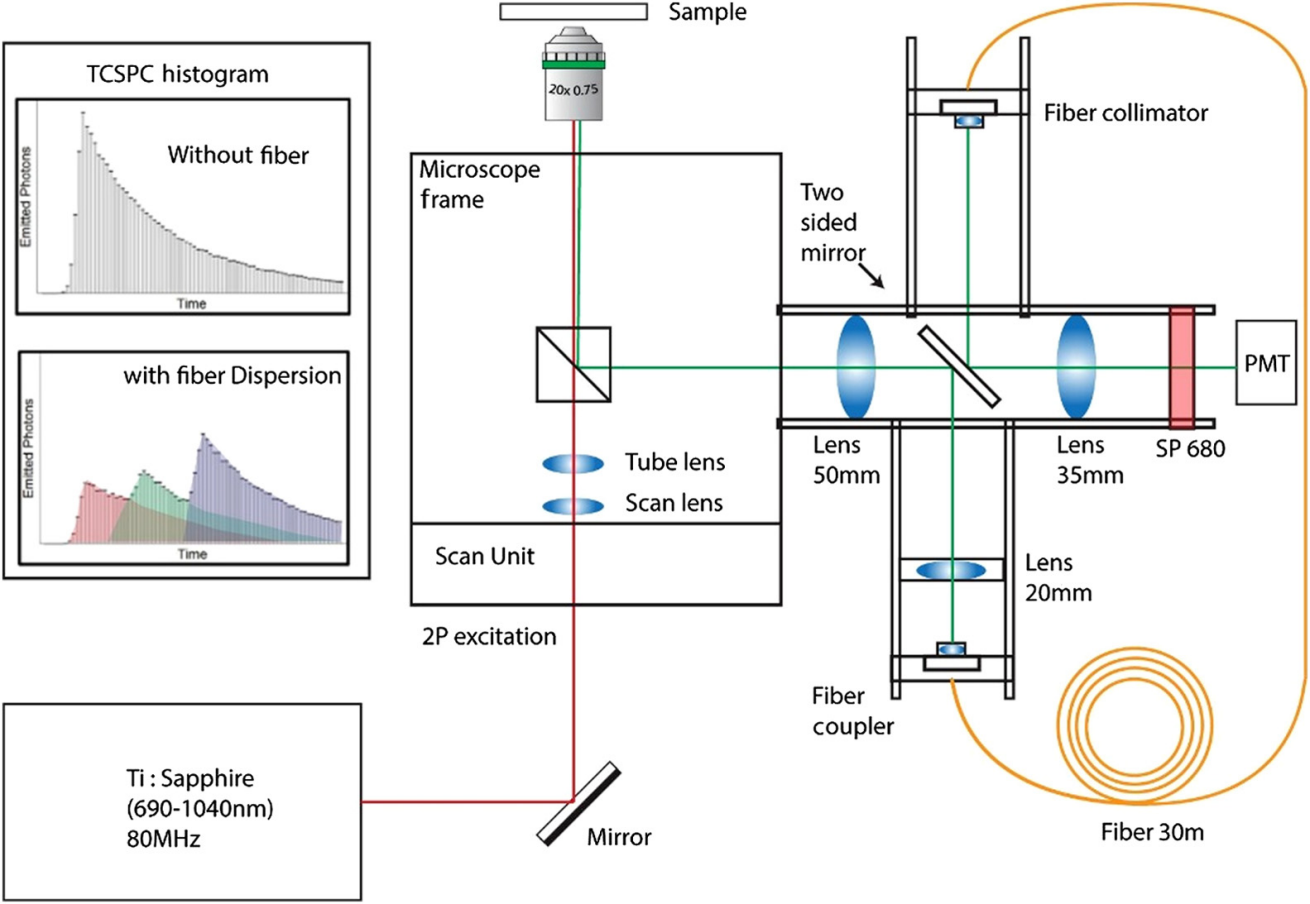
Schematic of the fiber-based spectral lifetime experimental setup. (PMT: photomultiplier tube, SP680: 680-nm shortpass filter). The figure inset TCSPC histograms show representative temporal spread in RGB wavelengths. Without the fiber, i.e., in conventional TCSPC setup, fluorescent decay curves for three separate fluorophores overlap and the contribution from the individual fluorophore is not evident (top). With the spectral separation introduced by the fiber, the different colored fluorophores are easier to distinguish, even without deconvolution (bottom).

A schematic of our experimental setup is shown in Fig. 2. This instrumentation is implemented on a custom-built multiphoton microscope built around an inverted Nikon microscope frame (Nikon Eclipse TE2000). The excitation source is a tunable ultrafast titanium:sapphire laser (Coherent Chameleon Ultra II) with a pulse repetition rate of 80 MHz. The imaging was carried out using a 20× air objective [Nikon, Plan Apo VC, numerical aperture (NA) = 0.75] and a 60x oil objective (Nikon, Plan Apo VC, NA=1.4). The emission beam splitters in the microscope frame allow one to direct the fluorescence emission into a custom-built side arm on a fixed optical cage assembly or to the regular imaging ports. A Uniblitz shutter (not shown in the schematic), which only opens during active image acquisition, is employed on the side port before the 50-mm lens to protect the detector from unintentional overexposure of light. The emission light is demagnified in beam diameter by a telescopic lens combination of plano-convex lenses (f=50  mm, Φ=1  in. and f=20  mm, Φ=1/2  in., respectively, Thorlabs N-BK7). This smaller beam diameter allows a better optical coupling to the fiber coupler (Thorlabs CFC-11X-A) with a built-in aspheric collimating lens (f=11  mm, NA=0.3, clear aperture Φ=6.6  mm).

The fiber coupler is mounted on a tiltable cage plate (Thorlabs KC1-S X/Y/Tilting cage plate) with adjustable axial distance and angle between the fiber tip and the collimating lens to aid in optical coupling. On the output end of the fiber, a fiber collimator (Thorlabs F810C-543) is used with a doublet lens (f=35  mm), followed by a condenser lens (f=35  mm) that focus the emission light onto a GaAsP photon counting PMT (Hamamatsu H7422P-40, Hamamatsu Photonics, Bridgewater, New Jersey). From the detector, photon data are time tagged using TCSPC electronics (SPC-150 Photon Counting Electronics, Becker & Hickl GmBH, Berlin, Germany). The fluorescence decay histogram is created within a 12.5-ns temporal duration defined by laser pulses (for 80-MHz laser). The duration is divided to 256 time-bins by an 8-bit time-to-digital conversion. Each time bin is ∼40  ps and each photon collected during the 12.5-ns acquisition window is placed in one of the time bins based on the arrival time with respect to the laser signal. The distribution of photons in the 256 time-bins essentially creates the fluorescence decay histogram. A 680-nm shortpass filter (Semrock FF01-680/SP-25) is placed in front of the PMT to block any residual multiphoton excitation light in the emission path. An additional feature of the experimental setup is the removable back-to-back mirrors (Thorlabs PFR10-P01) on the cage assembly (Thorlabs 30 mm). The mirrors can be easily removed, and then the collecting lens focuses the light from the microscope directly on the detector, bypassing the fiber. This allows convenient switching between spectral separation setup (with the mirrors) and conventional single-channel FLIM (without the mirrors). While this mirror is not essential to the experiment, it provides a convenient way to verify the performance of the system by imaging with and without the fiber. A challenge with this presented spectral lifetime fiber implementation is the efficient coupling of the uncollimated sample emission light into the fiber. Multimode fibers with large core diameters facilitate this coupling. This is discussed later in Sec. 4.1 on fiber—scan angle dependence.

### 3.2 Fibers in the Experiment

Primarily due to scattering, the propagation of visible light through the core of the optical fiber suffers from intensity loss (attenuation). The fluorescence emission spectra for commonly used fluorophores fall in the wavelength range of 400 to 700 nm, so fibers with lower attenuation in this range are required. Glass fibers are preferred over plastic ones owing to their lower attenuation.^32^ Based on manufacturer’s datasheets (Corning,^33^ Fujikura,^34^ and Thorlabs^35^), the attenuation of common off-the-shelf glass fibers are between 30 and 60  dB/km (∼66% to 81% transmission for 30-m length) at 445 nm. The transmission of the fibers listed in Table 1 were measured with a 445-nm laser. Modal dispersion was measured by sending a second-harmonic generation (SHG) signal through a fiber and measuring the rise time of the transmitted pulse (i.e., IRF) using Becker & Hickl TCSPC electronics. An SHG signal from a urea crystal (Sigma-Aldrich) was used rather than a fluorescence signal because it is a quasi-instantaneous process, which allows the IRF to be measured without the effects of fluorescence lifetime delays in the sample. The IRF is broadened by the modal dispersion and is also affected by the finite spectral bandwidth of the SHG radiation, when measured with a fiber.

**Table 1:** List of optical fibers used in the spectral lifetime experiment. Some data are unavailable from the manufacturers. Values in italic are measured by authors.

Fiber model	Type	Core material	Core diameter (μm)	Length (m)	NA	Attenuation (dB/km) at 445 nm	Measured transmission at 445 nm (%)
Corning Clear Curve OM4	GRIN	Silica	50	30	0.2	—	*71*
Corning InfiniCor300 OM1			62.5	10, 30, 50	0.275	—	*83/65/53*
Newport F-MLD-C		Unknown	100	30	0.29	—	*58*
Fujikura G.400/500		${\mathrm{GeO}}_{2}\text{-}{\mathrm{SiO}}_{2}/{\mathrm{SiO}}_{2}$	400	30	0.21	30	—
Fujikura G.600/750			600	30	0.21	30	—
Fujikura G.800/1000			800	30	0.21	30	—
Thorlabs FP400URT	Step index	Silica	400	10	0.5	30	*65*
Thorlabs FP400ERT			400	30	0.5	60	*61*
Thorlabs FP400URT			400	50	0.5	30	*45*

### 3.3 Sample Preparation

#### 3.3.1 Mixed fluorescence beads

The bead samples used in our experiments were mixed fluorescent microspheres (Polysciences, Inc.) that have distinct, well-characterized emission spectra. Two kinds of beads were used: Fluoresbrite^®^ 10-μm yellow–green (YG) carboxylate microspheres (Cat#18142-2,^36^ emission peak 486 nm) and Fluoresbrite^®^ 2.0-μm polychromatic red microspheres (Cat#19508-2,^37^ emission peak 565 nm). The mixture was applied to the surface of a glass microscope slide, dried, and then covered with a #1.5 coverslip, which was then sealed with nail polish. In each mixture, the two kinds of microspheres were chosen so that they share common excitation wavelengths and their distinct emission peaks were ∼80  nm apart. The measured lifetime value for YG beads was 2.45 ns and for red beads was 2.80 ns. Instrument response for TCSPC was measured as the SHG signal from urea crystals (Sigma-Aldrich).

#### 3.3.2 Cells

Fixed bovine pulmonary artery endothelial cells (BPAEC) purchased from ThermoFisher (Catalog #F36924) were prelabeled with: MitoTracker^®^ Red CMXRos for mitochondria, Alexa Fluor^®^ 488 phalloidin for F-actin, and 4′,6-diamidino-2-phenylindole (DAPI) for nuclei.

### 3.4 Data Analysis

The spectral information is extracted from the lifetime data by calculating the shift (i.e., delay) of the peak position of the exponential decay curve. Typical lifetime data analysis requires an IRF which is measured using SHG to estimate the excitation laser pulse’s position and the instrument timing resolution. Measured lifetime curves are convolved with this system response function. Decay fitting of lifetime curve uses a parameter called “shift” which is the timing difference of the peak of fluorescence decay to the excitation pulse due to the IRF. In the case of the data taken with the fiber, this shift of lifetime curve with respect to the IRF is dominated by the spectral shift. The wavelength-based delay (i.e., temporal chromatic dispersion) introduced by the fiber (∼20  ps/nm) for a 30-m-long fiber generates ∼400  ps for 20 nm spectral difference. The ideal IRF of a standard TCSPC FLIM system (in our case, Becker & Hickl electronics with a Hamamatsu PMT) is ∼200  ps. The IRF of our system gets broadened by optical components, such as fibers; the measured IRF with 30-m 800-μm core Fujikura graded-index (GRIN) fiber was ∼320  ps. Nevertheless, the ∼400  ps delay is still significantly larger than the measured IRF of our system and thus can be temporally resolved. Since the spectral shift produced by the fiber is a linear function of this measured shift in peak position [Fig. 6(c)], the spectral value per pixel may be determined by the shift of the peak of the decay curve measured for each pixel (each lifetime curve yields one wavelength). Practically, TCSPC works at the single-photon regime and a spatial binning can be used to aggregate the photons from neighboring pixels to reduce the error in estimating the peak of the decay curve. A 5×5  pixel binning can improve the accuracy of determining the center of shift by 5 times.

The workflow used to determine both lifetime and spectral distribution has three main steps. (1) Calculating shift from decay curve, (2) determining the fiber calibration factor (ratio of unit shift in wavelength to unit shift in time), (3) and mapping the shift from each pixel to emission spectral peaks based on this calibration factor using output of steps 1 and 2. Finally, an additional optional step would be deconvolution of IRF and estimation of lifetime. The lifetime data presented in the study use the lifetime estimation by mathematical fitting provided by the TCSPC analysis software SPCImage (Becker & Hickl GmbH). SPCImage offers a time-shift estimation along with the lifetime estimation. The shift parameter in SPCImage determines the temporal shift of the rising edge of the decay curve relative to the IRF (taken with fiber) position. Unfortunately, the SPCImage estimation of shift is coupled to the lifetime fitting algorithm and requires significant computational time. We wrote a code snippet that calculates the location of the peak of the lifetime decay independent of the fitting procedure and all the figures shown in this paper which displays delay measurement are generated using this method. This method is fast and can extract the delay information virtually instantaneously without the exponential curve fitting required by SPCImage to calculate shift. For the data that show lifetime in this paper, the lifetimes were calculated using SPCImage. For step 2, we measured the shift for a series of SHG wavelengths with the fiber and the calibration factor was calculated by a linear fitting of wavelength versus shift plot. For example, the calibration factor for converting temporal shift to emission spectrum for 800-μm 30-m Fujikura fiber was estimated to be 1.90 nm/(40-ps time-bin) in a 256-bin TCSPC collection scheme with a pulsed laser of 80-MHz repetition rate. Using these calibration data, the relative wavelengths (difference in the emission peaks with respect to the IRF position) for a lifetime image can be mapped to each pixel’s shift value. The shift image can also be adjusted using a custom calibration factor and/or a custom offset for adjusting the spectrum using a known maximum emission wavelength from a fluorescence dye.

Regions of interest (ROI) were created by segmenting the intensity images using ImageJ. For the bead images presented, the beads were separated by a common intensity image mask and filtered by a size criterion of beads as YG (diameter>6  μm) and red (diameter<4  μm) beads. This ROI-based separation splits the image visually into two groups and their respective distributions of shift are measured. For calculating relative shift between the two groups, we calculate the difference in mean values for both distributions. For the cell images, the background is filtered out where the photon counts are low, and the colors are separated by visual separation of the morphology.

## 4 Results

### 4.1 System Optimization

A longer optical fiber will give a better spectral resolution, but it will also suffer from larger signal attenuation. Fluorescence emission from biological samples has limited signal strength, so it is desirable to obtain a compromise between transmission efficiency and sufficient spectral separation. In this section, various optical-fiber-related factors are studied for their effects on the system performance.

#### 4.1.1 Fiber length

As discussed in Sec. 2, to achieve sufficient spectral separation in our proposed setup, the optical fiber should be on the order of 10 m or longer. A variety of “off-the-shelf” step-index and GRIN optical fibers of different lengths were evaluated in our system by imaging the same sample: a mixture of 10-μm YG (486-nm emission peak) and 2-μm red (565-nm emission peak) fluorescent beads with the same excitation wavelength (980 nm) and at the same ROI. This was made possible by the modular cage assembly and universal fiber couplers.

**Fig. 3:**
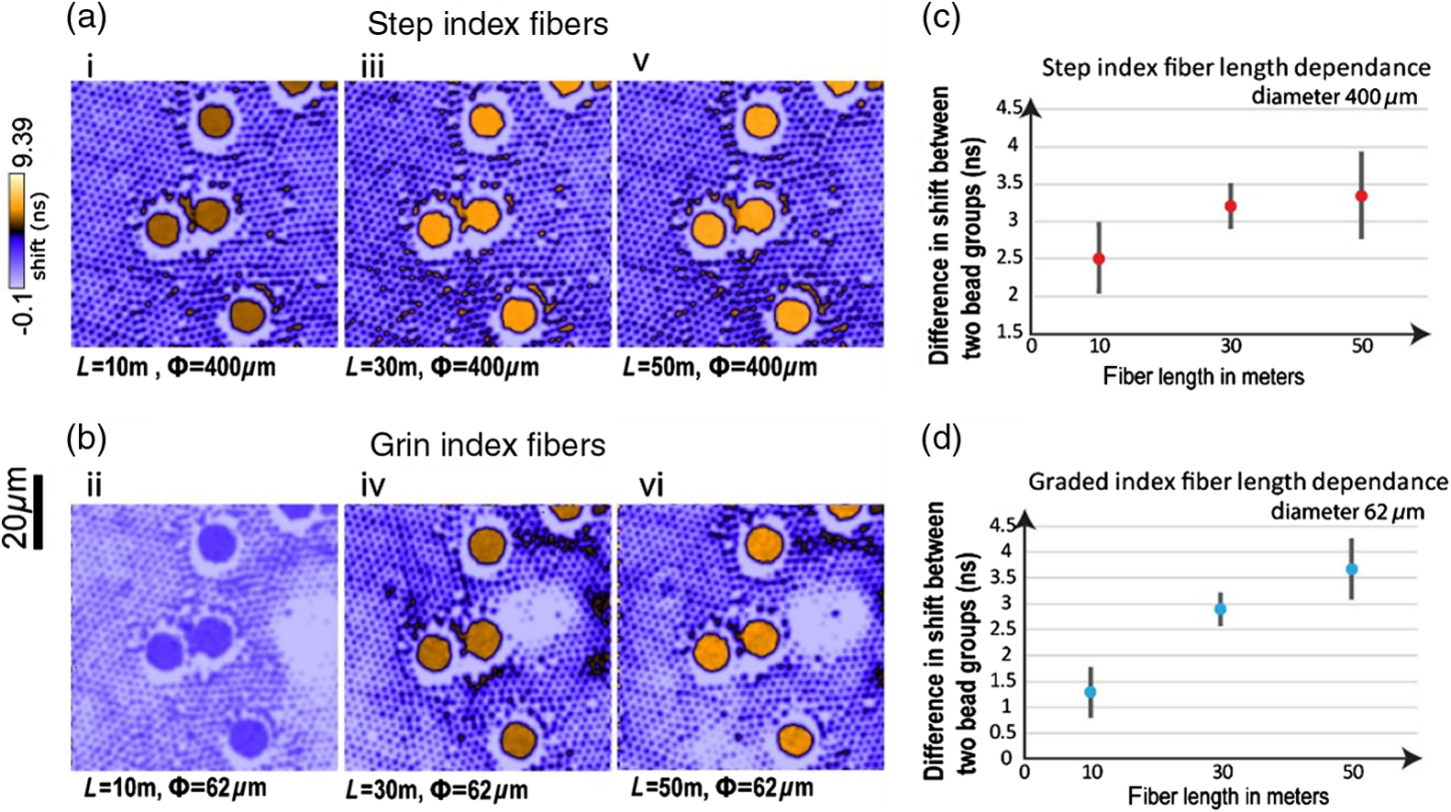
Spectral separation (represented by relative temporal shift in ns between two bead groups) demonstrated with step-index and GRIN fibers of various lengths. The fibers used are (a) Thorlabs 400/425-μm step-index fibers and (b) Corning InfiniCor300 OM1 62.5/125-μm GRIN fibers of 10, 30, and 50 m in length, respectively (refer to Table 1). The fiber length (in meters) and fiber diameter (in μm) are marked below each image. The sample is a mixture of 10-μm YG (emission 486 nm) and 2-μm red fluorescent microspheres (emission 554 nm) excited at 490 nm. (c) and (d) The difference in averaged shift between two bead groups for 400-μm step-index fibers and 62.5-μm GRIN fibers of three lengths. Images were taken with a 20× air objective (NA=0.75). The acquisition time was 60 s for all the images in this paper unless otherwise specified.

At the same ROI on the same sample, FLIM data were recorded with Thorlabs 400/425-μm step-index fibers and Corning InfiniCor300 OM1 GRIN fibers of 10, 30, and 50 m in length. The temporal delay (shift) at each pixel introduced by the chromatic dispersion of a fiber is extracted from fluorescence delay curves and plotted as colormap at the same scale for all fibers [Figs. 3(a) and 3(b)], using the method described in Sec. 3.4. The color contrast in the images represents the difference in the spectral distributions of two bead population (∼80  nm apart) translated in shift. In the shift images, higher color contrast between pixels corresponds to a larger relative temporal shift, thus resulting in a better spectral separation for different colored fluorophores in the same sample. The 10-m fibers can separate YG and red beads whose emission peaks are ∼80  nm apart; but longer fibers offer better spectral separations. The two 10-m GRIN and step-index fibers show significant differences between them. Although we have not examined this further, this is possibly due to differences in their composition and doping profiles.

Figures 3(c) and 3(d) show the difference in averaged shift between two bead ROIs (large YG beads and small red beads) for fibers of different lengths. The method was described in the last paragraph in Sec. 3.4. For both step-index fiber and GRIN fibers, we observe the same trend, i.e., the delay increases with fiber length. The relationship may not be linear in the experiment possibly due to the nonlinear dependence of the group velocity (also the temporal delay) on the wavelength, the error in spatial segmentation where pixel of the neighboring segment is incorrectly accounted for, and the error in estimating the peak positions of decay curves. However, an underlying linear relationship is possible as this would be within our experimental error bounds.

The collimator lens in front of the fiber has an NA=0.3. The Thorlabs step-index fibers have NA=0.5 and GRIN fibers have NA=0.275. Therefore, the modes in the GRIN fibers were filled at the fiber input but not those of the step-index fibers (although these can still be filled at the fiber output through a process called mode mixing or mode scrambling). Fibers were coiled in the same way as when they were shipped from the manufacturers, i.e., larger than the minimal suggested bending curvature.

#### 4.1.2 Fiber diameter and fiber type

Within the FOV of a laser scanning microscope, each pixel of the scan pattern in the sample plane represents an incident excitation at a particular galvanometer scan angle of the excitation beam on the back aperture of the objective lens. In our system (Fig. 2), the emission signal from all of the pixels (i.e., scan angles) is relayed to the tip of the fiber for photon collection with the help of the additional optics on the side arm (f=50  mm and f=20  mm plano-convex lenses and the f=11  mm collimator lens). The maximum FOV (i.e., at zoom 1) is achieved with a galvanometer scan angle of ±3  deg. The excitation light is relayed to the objective using a scan lens and a tube, with the scan angle scaled down to ∼±1  deg after the tube lens. Therefore, the input scan angle to the side arm of the microscope on the left side of the 50-mm lens is ±1  deg at zoom 1. The NA is 0.09 at the output of the microscope frame and 0.3 at the input to the fiber.

**Fig. 4:**
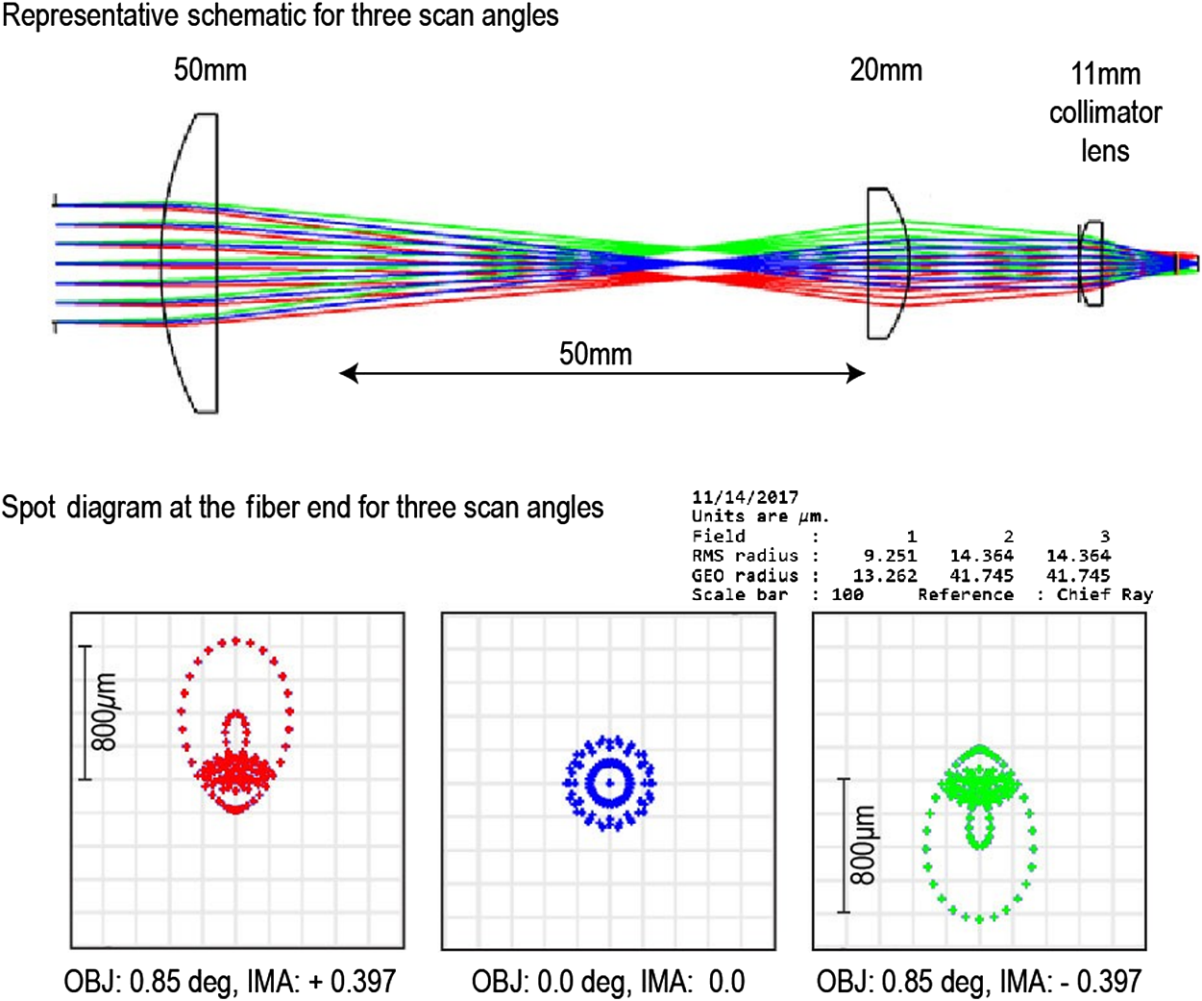
Zemax simulation of the fiber coupling in the proposed system to study the theoretical limits of the transmission. The combination of f=50  mm (diameter of 1 in.) and f=20  mm (diameter of 1/2 in.) plano-convex lenses is used to reduce the beam diameter of fluorescent emission light, which is in turn focused to the tip of the fiber which is mounted to Thorlabs CFC-11x-A collimator by a built-in f=11  mm aspheric lens (NA=0.3). At ±0.85-deg input scan angle to f=50  mm lens, the FOV at the tip of the fiber is 800  μm, matching the core diameter of Fujikura G.800/1000 GRIN fiber. Different colors represent different scan angles.

The fiber coupling scheme of our current experimental setup was simulated with Zemax software (Fig. 4). Assuming that the emission signal is perfectly focused at the tip of the fiber (i.e., minimizing the RMS radius of the focused beam), it has 930-μm FOV at the back focal plane of the collimator lens at nominal scan angle (±1  deg on the left side of the 50-mm lens at zoom 1), leading to 74% ${\left(800/930\;\mu m\right)}^{2}$ transmission coefficient. By reducing the scan angle, i.e., higher zoom, the FOV at the back focal plane of the collimator lens decreases, resulting in improved transmission ratio. The Zemax simulation shows that when the scan angle is <0.85  deg, the FOV at the tip of the fiber will be <800  μm, a size that matches the core diameter of Fujikura G.800/1000 GRIN fiber (Table 1), leading to optimal coupling. Therefore, it is evident that the core diameter of the fiber selected should be as large as possible to allow for efficient coupling of emission signals from large scan angles.

Note that in our experiment, we intentionally defocus the incident beam at the tip of the fiber to allow the signal from large scan angles that would otherwise be focused outside the fiber core to be partially collected by the fiber, effectively increasing the FOV of the system, although at the cost of reduced transmission efficiency as well as more vignetting. It is especially helpful when we want to obtain a large FOV with small core fibers.

**Fig. 5:**
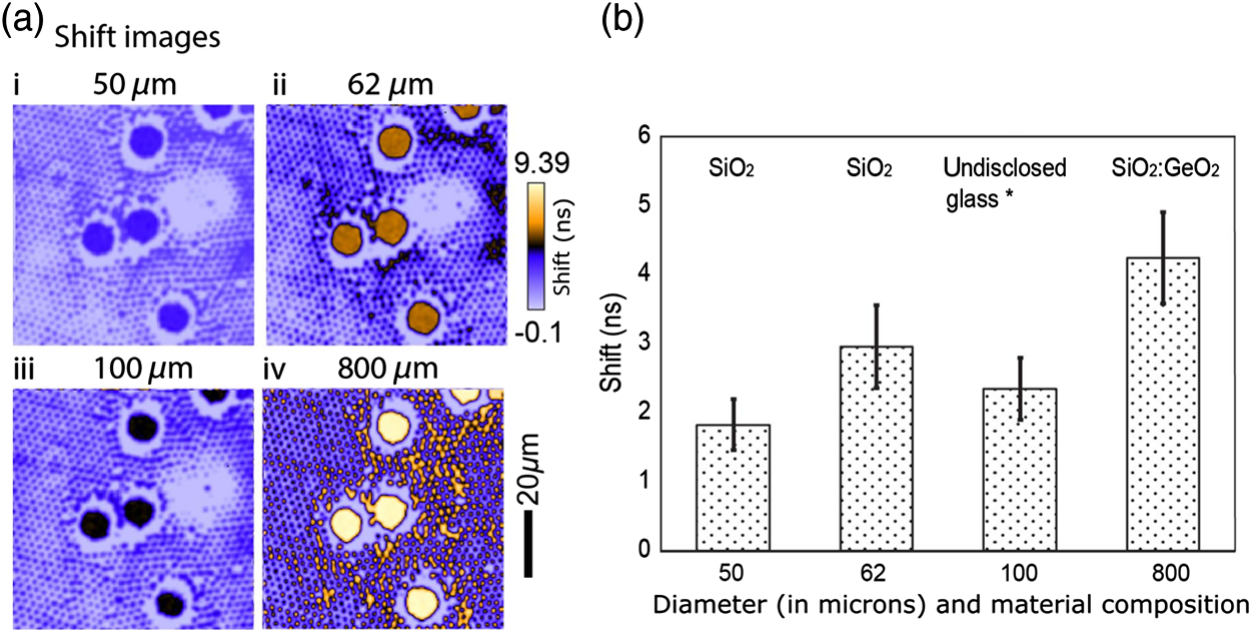
Spectral separation (translated to relative temporal shift in ns) demonstrated with 30-m-long GRIN fibers of various core diameters and material compositions. (a-i) Corning ClearCurve OM4, (a-ii) Corning InfiniCor300 OM1, (a-iii) Newport F-ML-D-C, and (a-iv) Fujikura G.800/1000. (a) Shift images are plotted with the same look up tables (LUTs) for all the fibers. The sample is a mixture of 10-μm YG (emission 486 nm) and 2-μm red fluorescent microspheres (emission 554 nm) excited at 490 nm. (b) The difference in shift between two bead groups for different fibers. Images were taken with a Nikon Plan Apo 20×/0.75 objective. In this experiment, 8x zoom was used to achieve the same FOV for all the fibers for a fair comparison.

The impact of fiber diameter and fiber material on spectral separation was also studied (shown in Fig. 5) with four types of GRIN fibers: Corning ClearCurve OM4, Corning InfiniCor300 OM1, Newport F-ML-D-C, and Fujikura G.800/1000, with core diameters of 50, 62.5, 100, and 800  μm, respectively. From the images of relative temporal delays [the second column in Fig. 5(a)], clearly Fujikura 800-μm core GRIN fiber performs best in spectral separation, possibly because of its core material and doping profile, with the Corning 62.5-μm GRIN fiber second in place. The same trend can also be revealed quantitatively by the difference in averaged delay between the bead groups [Fig. 5(b)]. We found no scan angle dependence on the shift over the entire FOV for all the fibers. Note that 400- and 600-μm core Fujikura fibers generated similar spectral separation as the 800-μm one. Considering that larger core diameter provides better coupling, we showed only the 800-μm result in Fig. 5.

Overall, for sufficient spectral separation and maximum transmission efficiency, we chose the 30-m multimode GRIN glass fiber with the largest possible core diameter (>800  μm) and the largest available NA (0.21). Thus, out of the fibers that we are in possession of (Table 1), the 30-m Fujikura 800/1000-μm GRIN fiber appears to the most practical candidate for the spectral lifetime system.

### 4.2 Spectral Mapping

Experiments with different fibers using the same sample, FOV, and similar excitation wavelength revealed the best delay and transmission properties among the fibers (Figs. 3 and 5). As expected, longer fibers provide better spectral contrast and fibers with larger core diameter and lower attenuation increase the signal-to-noise ratio. The contrast obtained by the 30-m 800-μm GRIN fiber was higher than other fibers and was chosen for the setup. With the optimized setup described above, a mixture of YG:red beads was imaged to characterize the spectral and lifetime differences.

#### 4.2.1 Effect on lifetime estimates because of the fiber

**Fig. 6:**
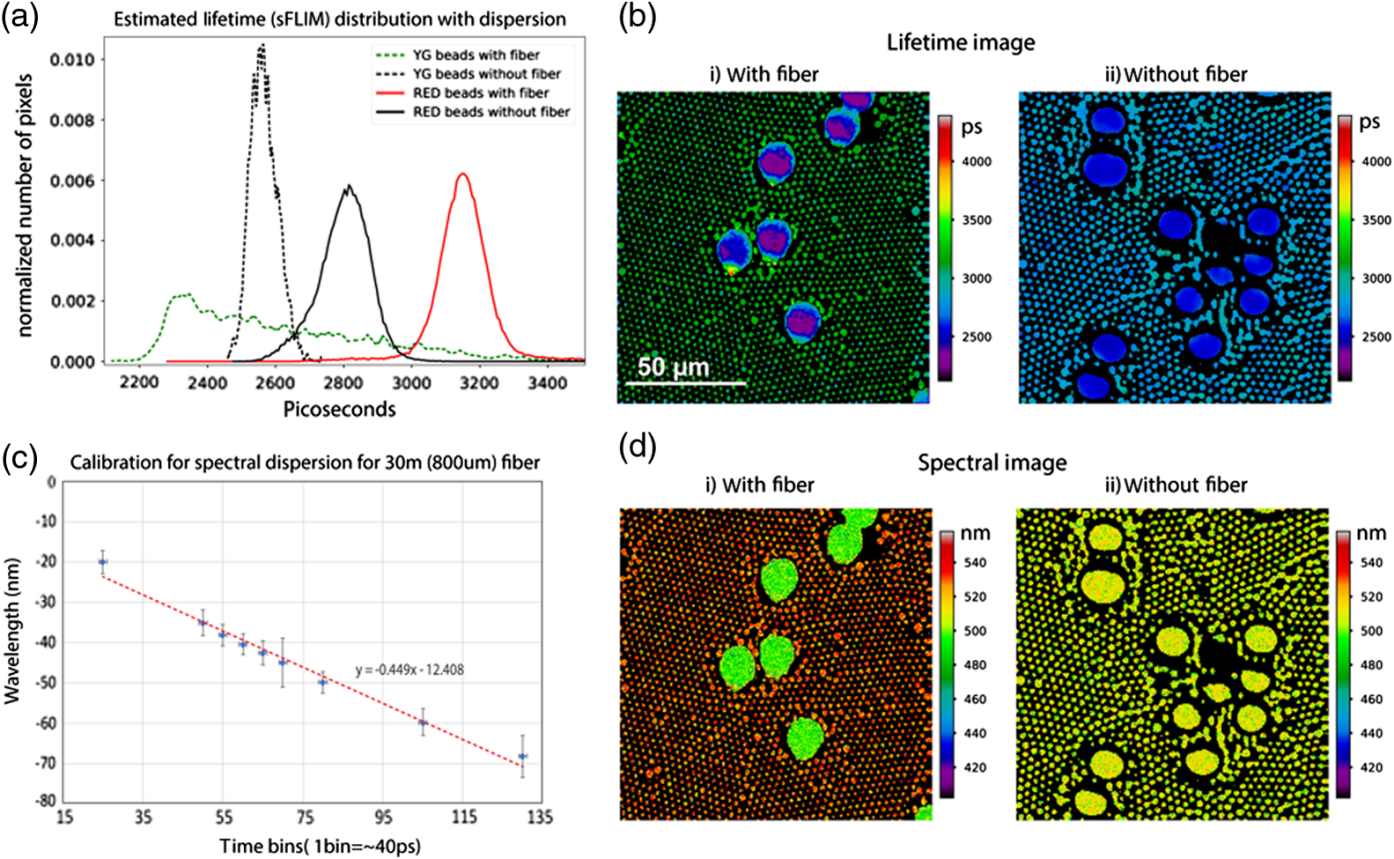
(a) Estimated lifetime distribution with fiber dispersion for YG:red bead mixture. The averaged estimated lifetime for YG shifts from 2.2 to 2.1 ns and for red shifts from 2.8 to 3.18 ns. These delays (−0.1  ns) and (+0.3  ns) are an effect of the convolved spectral distribution with the real lifetime distribution of the observed photons. The colored curves are separable because of the fiber spectral dispersion. The green dotted line shows the YG beads and red dotted line shows the red beads with the fiber. The noncolored curves show the beads without fiber. (b) The estimated lifetime images in the same color scheme, with the color scale determined by the range of the estimated lifetime with fiber (2100 to 4500 ps). With the fiber, the dynamic range (thus, the contrast) of the image (b-i) increases compared to the (b-ii) nonfiber case. (c) Calibration curve for temporal delay due to fiber chromatic dispersion with respect to the change in the emission wavelength. For this calibration, second harmonic signal was measured for a series of wavelengths with the 30-m 800-μm GRIN fiber and used to create spectral LUT for the images. (d) Spectral images (obtained by mapping shift to spectrum) of the same measurements from (b) shown in the same spectral color scale. With the fiber, the red and YG beads can be visually identified in color, but there is no contrast and no meaningful spectral information is displayed.

Fluorescence lifetime estimation from TCSPC histograms is a straightforward mathematical parameter estimation under known physical constraints. Estimating the lifetime with the YG:red mixture is detailed in Sec. 3.4 and the results are shown in Fig. 6(a). A separation in the lifetime distribution is obtained from YG beads (∼2.55  ns) and red beads (∼2.80  ns) without the fiber. The broader distribution of the YG beads can be attributed to the spatial/temporal binning associated with the calculation of the shift near the edges of the beads. However, with the fiber in the emission path, the shift per pixel from the TCSPC photon distribution will be convolved with the sample exponential decay, and the estimated lifetime will diverge from its absolute lifetime. Figure 6(a) shows that the shorter lifetime of YG beads gets shorter and the longer lifetime of red beads gets longer. This is an effect of convolved spectral information on the lifetime decay curve. We use this increased contrast of the lifetime measurement to distinguish species in a lifetime—spectral domain. However, it should be noted that although the “with fiber” lifetime image produces the shift needed for estimating the spectral emission image, the resultant decay is the result of the TCSPC decay being convolved with the shift per pixel. Because fluorescence emission exhibits an extended spectrum rather than a single spectral line, there is a resultant shift of the measured mean lifetime value. For optimum result, a “without fiber” image is needed for the real lifetime distribution.

The YG and red beads with the fiber show estimated lifetimes (sFLIM) of 2.2 and 3.18 ns, a shift of −0.1 and +0.30  ns, respectively. As shown in Fig. 6(b), the range of the color values shown (contrast) in the fiber FLIM image is higher than that without the fiber. The LUTs are same for both panel Figs. 6(b-i) and 6(b-ii) and a visual comparison of two panels shows that the image with fiber spans a larger range of colors.

Note that the absolute lifetime determination does get complicated with conventional TCSPC fitting with the presence of the fiber. A complicated spectral-IRF deconvolution is required to get absolute lifetime values. A deconvolution of the lifetime with its spectral width can be done using spectral width estimation using pixel binning or using a time lapse to generate enough points to build a spectrum and find the spectral width. This approach will be pursued in future studies.

#### 4.2.2 Calibrating spectral separation per delay time bin

As described in Sec. 3.4, in order to calibrate the spectral separation obtained per time bin of TCSPC excitation for the 30-m fiber, the second-harmonic signal for a series of excitation wavelengths was measured and plotted against their corresponding delays to estimate the fiber calibration factor as −1.9  nm/(40 ps time-bin) (or −47.6  nm/ns) [Fig. 6(c)]. Based on the calibration factor, temporal shift images can be mapped and colored according to their mean spectral shifts. This color map was applied to the shift image to make spectral image as shown in Fig. 6(d). Figure 6(a) shows the estimated distribution of lifetime for both YG and red beads with and without the fiber. The lifetime for YG beads shifts by −0.1  ns and for red beads it is shifted by +0.3  ns. These delays can be attributed to the effect of convolution of real lifetime distribution with the spectral distribution. Each pixel has a whole spectral emission distribution which is convolved with the lifetime distribution of the individual fluorophores. This results in the deviation from the real lifetime distribution. Chromatic dispersion in fiber [Fig. 6(d-i)] is illustrated by the color-coded image, in which the two bead populations are clearly distinct. The image without fiber [Fig. 6(d-ii)] serves a comparison, showing the relatively small distribution in values that results from fluctuations in the measurement.

### 4.3 Spectral Mapping in Cells

The contrast demonstrated in the fiber-based sFLIM imaging can be used to distinguish different species and can address biologically important heterogeneity in samples. For spectrally separable populations, this approach helps to obtain a lifetime-independent degree of separation derived from the respective shift values.

#### 4.3.1 Spectral image

**Fig. 7:**
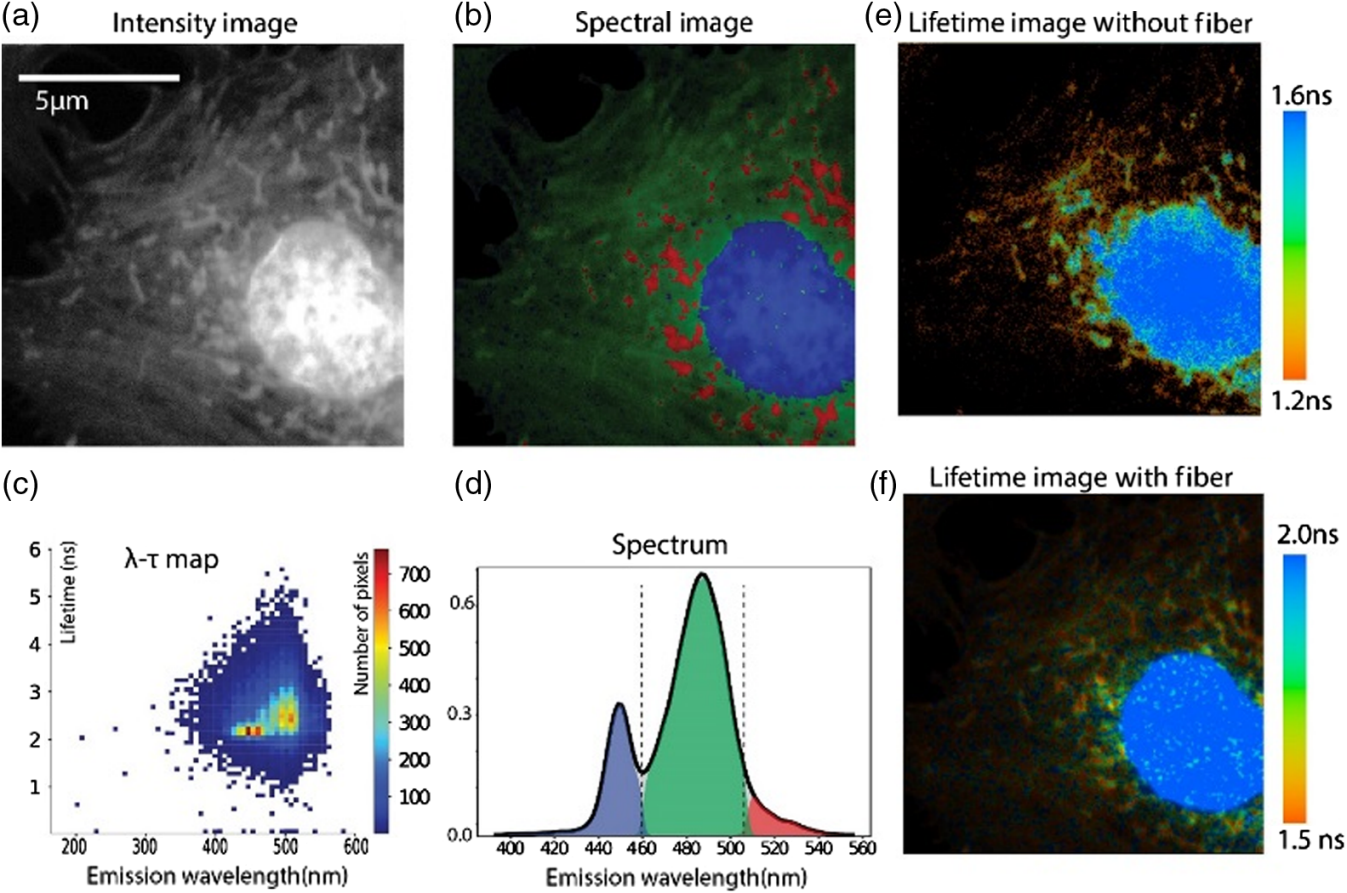
Hyperspectral image: Fluocells (ThermoFisher Scientific) prepared slides shows bovine pulmonary cells that are labeled with MitoTracker^®^ Red for mitochondria, Alexa-Fluor-488 for F-actin and DAPI. (a) Intensity image when excited with 750-nm multiphoton excitation. (b) Spectral image obtained by thresholding shift into 400 to 460 nm (blue), 460 to 507 nm (green), and 507 nm to Inf (red). (c) Two-dimensional histogram plotted between wavelength (calculated with fiber) and fluorescence lifetime (calculated without fiber). (d) Experimental result of spectral separation obtained from the spectrum (solid black line) into three colors, blue, green, and red, at the thresholding limits mentioned before (460 and 507 nm). Images were taken with a 60× oil objective (NA=1.4). (e) Lifetime image without fiber and (f) lifetime image with fiber (lifetime scale shown in the right).

To study the efficacy of this strategy of spectral separation, we procured prelabeled slices of BPAEC (FluoCells, ThermoFisher Scientific, Waltham, Massachusetts; Cat #F36924). The slides are labeled with MitoTracker^®^ Red CMXRos, Alexa-Fluor-488 phalloidin, and DAPI. This will give three channel color images showing red mitochondria, green F-actin, and blue DAPI. The sample was imaged under 750-nm excitation with the 800-μm 30-m GRIN fiber and the results are presented in Fig. 7. An intensity-adjusted image is shown in Fig. 7(a). The intensity adjustment is necessary because the DAPI is very bright under a common multiphoton excitation for the three fluorescent probes reported. The shift was calculated with the calibration used for the fiber and a custom offset, so that the DAPI spectrum peaks at the expected value of 450 nm. The software supplied has the capability to shift the spectrum measured to any known spectral peak instead of the IRF to offset the spectrum using a custom shift. For example, we provided a custom shift value of 450 nm for DAPI signal instead of the IRF measurement at 890 nm (445 nm). The value would be different for a different fiber-based setup. The spectrum was split into three based on two spectral thresholds at 460 and 507 nm to separate three cellular areas visually. The kernel density estimation plot for the spectral separation of the entire image (solid black curve) to the three colors is shown in Fig. 7(d). The calibration curve shown in Fig. 6(c) was used to create the distribution by converting the time shift to wavelength. This spectral separation can be used to color the intensity image as seen in Fig. 7(b). The histogram of lifetime (calculated without the fiber) and the spectrum calculated with the fiber is plotted in Fig. 7(c) as λ(wavelength) − τ(lifetime) plot. This histogram shows the separation of species better than either lifetime or spectrum alone. The colors can be separated as three species in this histogram using cursors or simply by a projection to the λ axis as shown in Fig. 7(d). Figures 7(e) and 7(f) show the lifetime images without and with the fiber, respectively. For calculating the lifetime for both of the images, SPCImage was used for performing the curve fitting.

## 5 Discussion

In this paper, a low-cost spectral add-on to an existing FLIM implementation is demonstrated. Utilizing an optical fiber added to the emission path of a time-domain FLIM multiphoton microscope, we achieve spectral discrimination in the FLIM signal. The technique exploits the fiber-induced chromatic dispersion of the fluorescence photon traveling through the fiber to identify its wavelength. Note that this method is not presented as an alternative to multidetector/filter systems,^23^ but a fast and cost-effective way to induce spectral contrast into an existing FLIM acquisition system. The spectral information will be coded as the rather larger time delay on the lifetime curve (50-nm spectral separation codes as 1-ns shift of the lifetime curve). Employing a method as demonstrated to acquire lifetime data both with and without fiber allows one to get both average lifetime and average spectral wavelength from a pixel, then building an FLIM and spectral image.

Chromatic dispersion is a function of the length of the fiber, and longer fibers increase the time delay between spectral components enhancing spectral resolution. However, longer fibers also introduce higher attenuation as well as larger modal dispersion, which limits the ability to resolve spectral separation and causes signal loss and reduces the signal-to-noise ratio. To make the system practical for investigating emission spectra of biological samples, the fiber should be long enough to produce separation with minimized modal dispersion, have a diameter large enough to accommodate the scanning light that is focused on the fiber tip, and be made of a material with an acceptable attenuation in the visible spectral range. Based on our experiments with our limited selection of optical fibers, we have found that using a 30-m-long 800-μm core diameter GRIN fiber from Fujikura Corporation^34^ gives the best result for spectral separation between species with the optimum transmission. With the current fiber-based spectral lifetime setup, we were able to spectrally separate and map fluorescent microspheres, using the calibrated 1.9  nm/40  ps time-bin (47.6  nm/ns) factor for converting emission spectrum shift into temporal shift measured in our TCSPC collection scheme.

*A priori* knowledge of lifetime or spectrum could be used to deconvolve the curve to get one or the other. All the data we collected have both lifetime and spectral information collected sequentially, but we have not explored any deconvolution methods in this study. While this system is not a substitute for previously published schemes, such as multichannel/multidetector^16^ or multichannel/single detector schemes,^23^ this method offers an alternative low-cost spectral discrimination scheme to a microscope.

For a simultaneous spectral lifetime acquisition and analysis, the calculated lifetimes with fibers diverge from the true lifetime values, due to the convolution between the lifetime and spectrum distribution. Future developments could be to acquire the ability to quantify the presence of various fluorophores in the same pixel or ROI using mathematical modeling of the temporal distribution of photons traveling through fibers. In the cases where known fluorophores with known spectra are used (which is commonly the case of biological labeling experiments), it should be possible to deconvolve the spectral and lifetime data. For estimating the lifetime faster, alternative methods, such as rapid lifetime estimation, can generate the mean lifetime faster from these images than multiparametric fitting. However, even without exploring this, we have demonstrated that the combined temporal/lifetime data can provide better discrimination between fluorescence signals rather than spectra or lifetime alone. Although it should be noted that, to identify multiple fluorophores, a custom offset is needed for a known fluorophore (specific to the fiber) relative to which the other fluorophores are identified.

To improve the resolution of the system, a detector free from afterpulsing and shortened IRF width could be used. Using a hybrid detector^38^ instead of a GaAsP detector should result in a better approximation of the IRF because of its lack of afterpulsing and narrow width resulting in a clean response function. We expect this to result in an improved temporal resolution.

In theory, a longer fiber should give us improved spectral resolution, but modal dispersion and fiber attenuation place practical limits to the use of longer fibers. As many commercially available fibers are not characterized for refractive index, modal dispersion, and attenuation in the visible spectrum, better characterization of the fibers in appropriate spectral ranges would help us to have a quantitative understanding of the potential performance and limitations of the approach, thus enabling us to optimize the system. The accuracy of the temporal delay calculation is limited by the IRF of the system. For current commercial TCSPC system, the photon detection accuracy is in the range of 100 to 350 ps with a timing resolution of ∼40  ps. The fitting routine also needs to be faster with better delay estimation for each pixel to account for the spectral data being convolved with the lifetime data.

The long-term goal of this system is to identify multiple fluorophores accurately with improved resolution from a single acquisition and utilize this new combined lifetime and spectral modality to solve biological challenges. This study provides the foundational work for such a faster multimodal acquisition scheme.
